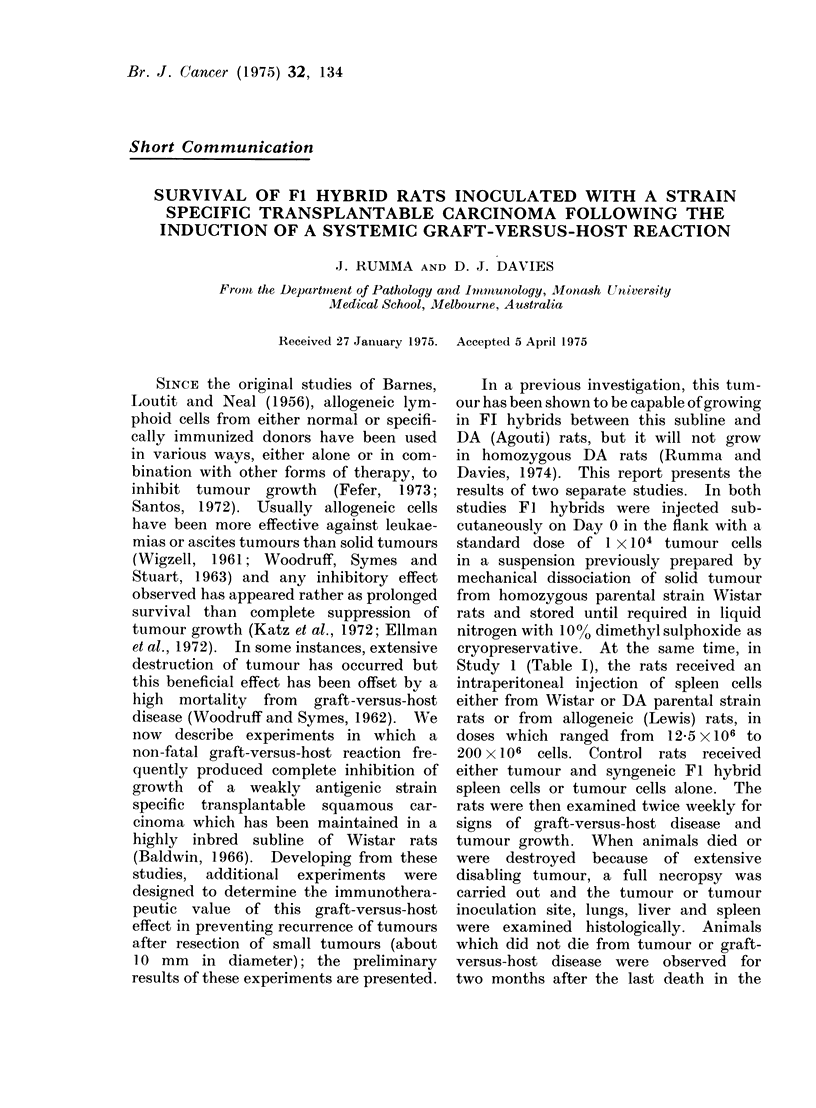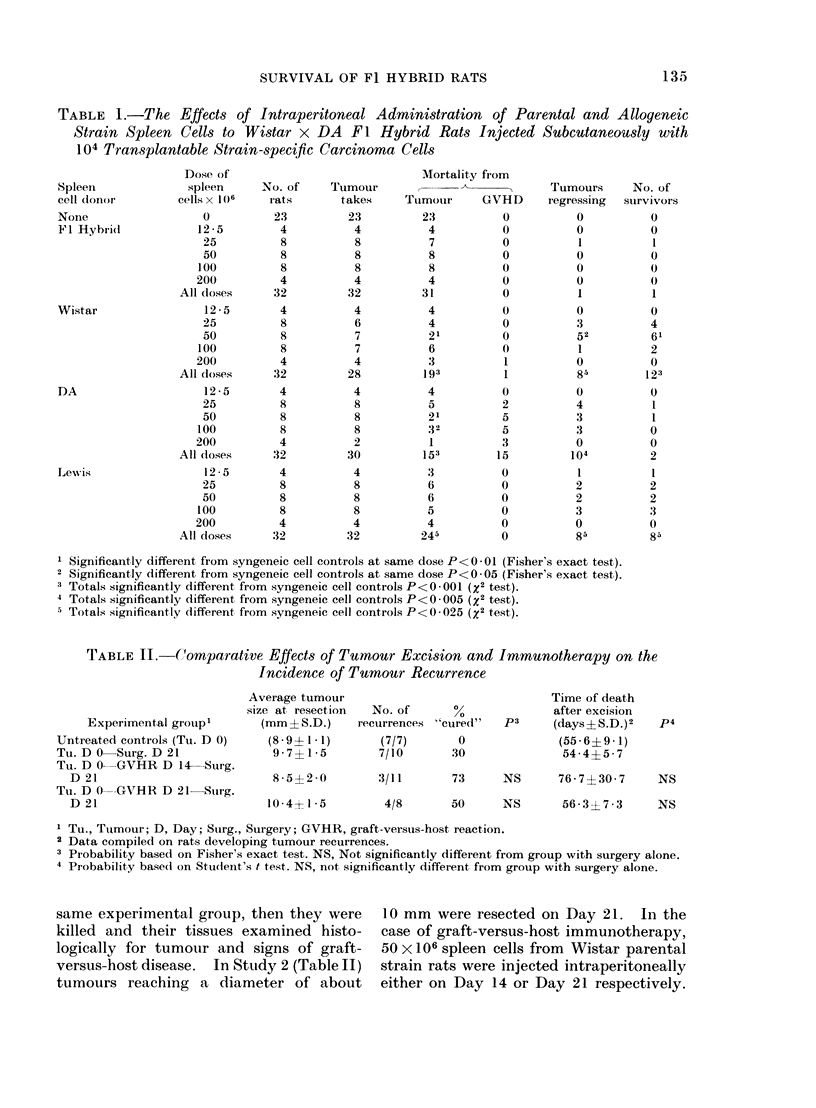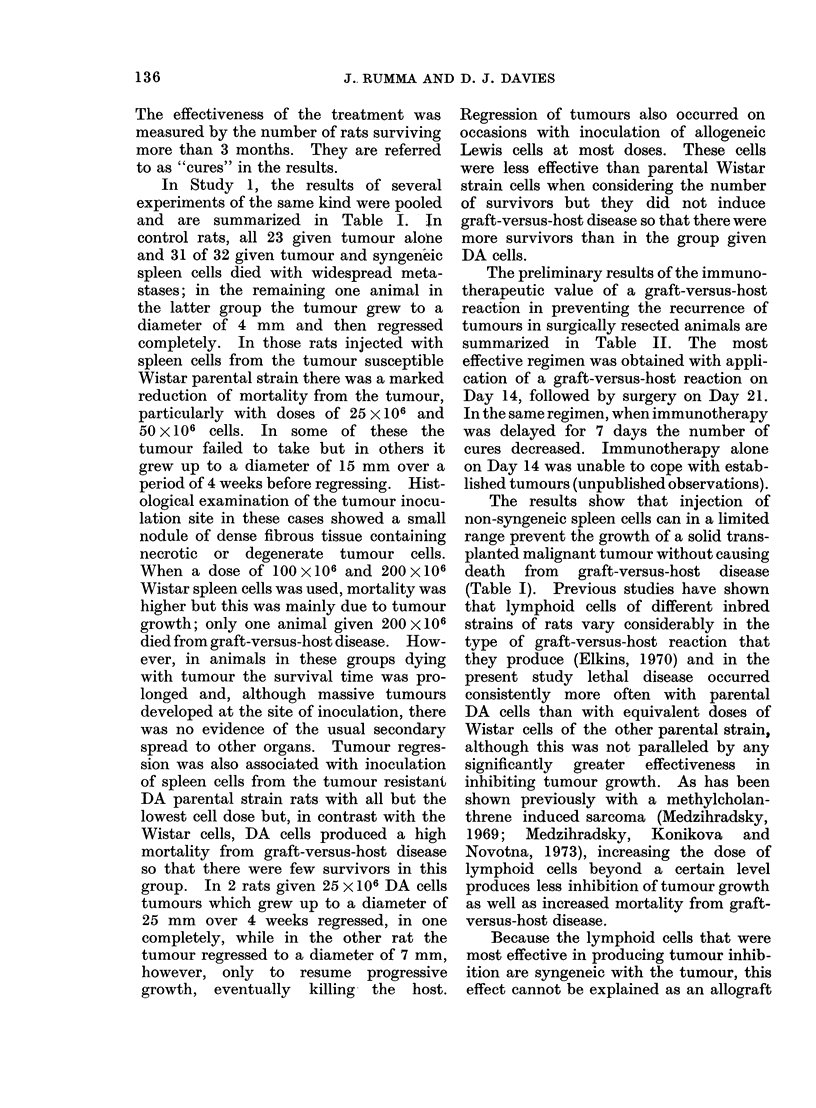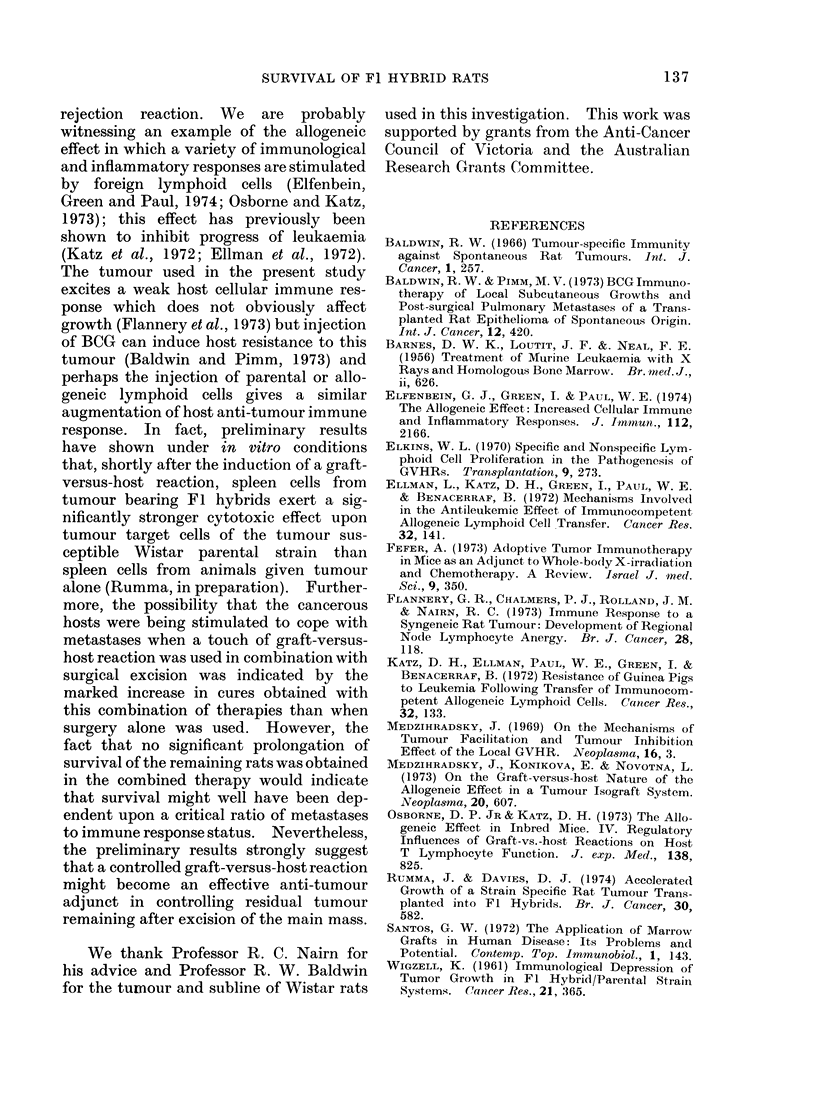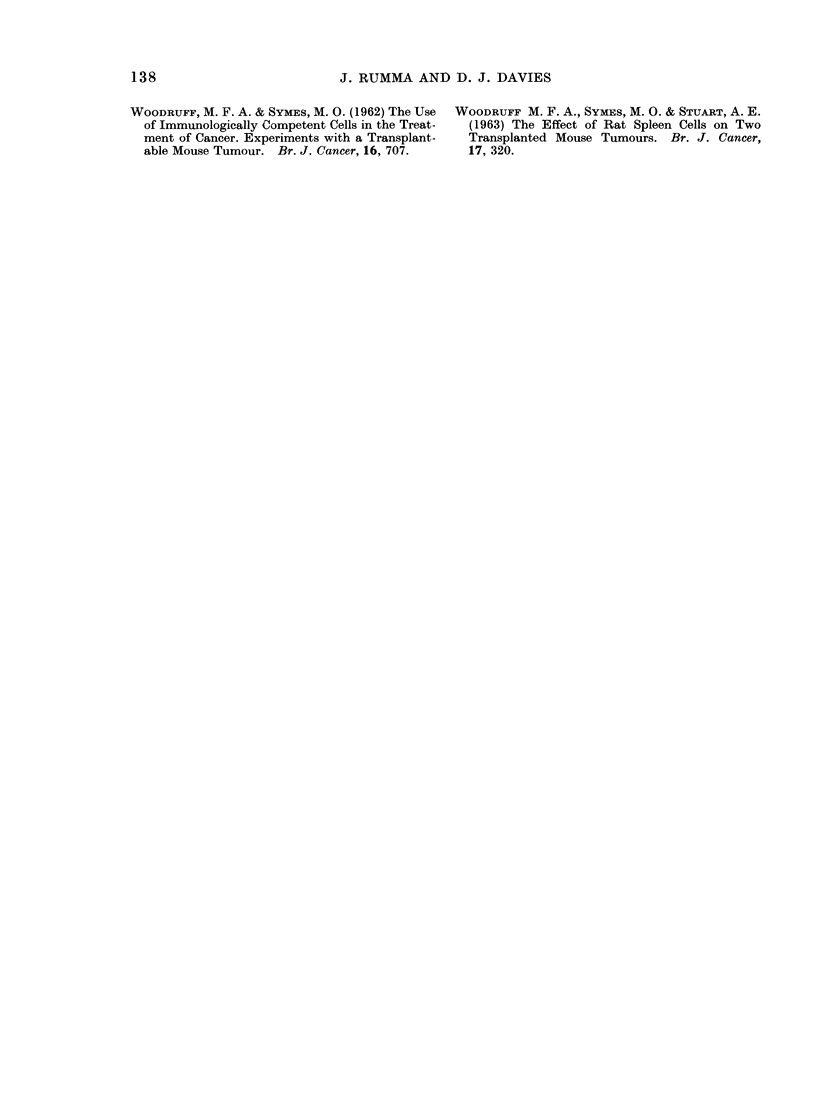# Survival of F1 hybrid rats inoculated with a strain specific transplantable carcinoma following the induction of a systemic graft-versus-host reaction.

**DOI:** 10.1038/bjc.1975.139

**Published:** 1975-07

**Authors:** J. Rumma, D. J. Davies


Br. J. Cancer (1975) 32, 134

*Short Communication*

### SURVIVAL OF F1 HYBRID RATS INOCULATED WITH A STRAIN SPECIFIC TRANSPLANTABLE CARCINOMA FOLLOWING THE INDUCTION OF A SYSTEMIC GRAFT-VERSUS-HOST REACTION

J. RUMMA AND D. J. DAVIES

*From the Department of Pathology and Immunology, Monash University Medical School, Melbourne, Australia*

Received 27 January 1975. Accepted 5 April 1975

SINCE the original studies of Barnes, Loutit and Neal (1956), allogeneic lymphoid cells from either normal or specifically immunized donors have been used in various ways, either alone or in combination with other forms of therapy, to inhibit tumour growth (Fefer, 1973; Santos, 1972). Usually allogeneic cells have been more effective against leukaemias or ascites tumours than solid tumours (Wigzell, 1961; Woodruff, Symes and Stuart, 1963) and any inhibitory effect observed has appeared rather as prolonged survival than complete suppression of tumour growth (Katz *et al.*, 1972; Ellman *et al.*, 1972). In some instances, extensive destruction of tumour has occurred but this beneficial effect has been offset by a high mortality from graft-versus-host disease (Woodruff and Symes, 1962). We now describe experiments in which a non-fatal graft-versus-host reaction frequently produced complete inhibition of growth of a weakly antigenic strain specific transplantable squamous carcinoma which has been maintained in a highly inbred subline of Wistar rats (Baldwin, 1966). Developing from these studies, additional experiments were designed to determine the immunotherapeutic value of this graft-versus-host effect in preventing recurrence of tumours after resection of small tumours (about 10 mm in diameter); the preliminary results of these experiments are presented.

In a previous investigation, this tumour has been shown to be capable of growing in FI hybrids between this subline and DA (Agouti) rats, but it will not grow in homozygous DA rats (Rumma and Davies, 1974). This report presents the results of two separate studies. In both studies F1 hybrids were injected subcutaneously on Day 0 in the flank with a standard dose of $1 \times 10^4$ tumour cells in a suspension previously prepared by mechanical dissociation of solid tumour from homozygous parental strain Wistar rats and stored until required in liquid nitrogen with 10% dimethyl sulphoxide as cryopreservative. At the same time, in Study 1 (Table I), the rats received an intraperitoneal injection of spleen cells either from Wistar or DA parental strain rats or from allogeneic (Lewis) rats, in doses which ranged from $12 \cdot 5 \times 10^6$ to $200 \times 10^6$ cells. Control rats received either tumour and syngeneic F1 hybrid spleen cells or tumour cells alone. The rats were then examined twice weekly for signs of graft-versus-host disease and tumour growth. When animals died or were destroyed because of extensive disabling tumour, a full necropsy was carried out and the tumour or tumour inoculation site, lungs, liver and spleen were examined histologically. Animals which did not die from tumour or graft-versus-host disease were observed for two months after the last death in the

TABLE I.—*The Effects of Intraperitoneal Administration of Parental and Allogeneic Strain Spleen Cells to Wistar × DA F*1 *Hybrid Rats Injected Subcutaneously with* $10^4$ *Transplantable Strain-specific Carcinoma Cells*

| Spleen cell donor | Dose of spleen cells × $10^6$ | No. of rats | Tumour takes | Mortality from Tumour | Mortality from GVHD | Tumours regressing | No. of survivors |
|---|---|---|---|---|---|---|---|
| None | 0 | 23 | 23 | 23 | 0 | 0 | 0 |
| F1 Hybrid | 12·5 | 4 | 4 | 4 | 0 | 0 | 0 |
| | 25 | 8 | 8 | 7 | 0 | 1 | 1 |
| | 50 | 8 | 8 | 8 | 0 | 0 | 0 |
| | 100 | 8 | 8 | 8 | 0 | 0 | 0 |
| | 200 | 4 | 4 | 4 | 0 | 0 | 0 |
| | All doses | 32 | 32 | 31 | 0 | 1 | 1 |
| Wistar | 12·5 | 4 | 4 | 4 | 0 | 0 | 0 |
| | 25 | 8 | 6 | 4 | 0 | 3 | 4 |
| | 50 | 8 | 7 | 2[1] | 0 | 5[2] | 6[1] |
| | 100 | 8 | 7 | 6 | 0 | 1 | 2 |
| | 200 | 4 | 4 | 3 | 1 | 0 | 0 |
| | All doses | 32 | 28 | 19[3] | 1 | 8[5] | 12[3] |
| DA | 12·5 | 4 | 4 | 4 | 0 | 0 | 0 |
| | 25 | 8 | 8 | 5 | 2 | 4 | 1 |
| | 50 | 8 | 8 | 2[1] | 5 | 3 | 1 |
| | 100 | 8 | 8 | 3[2] | 5 | 3 | 0 |
| | 200 | 4 | 2 | 1 | 3 | 0 | 0 |
| | All doses | 32 | 30 | 15[3] | 15 | 10[4] | 2 |
| Lewis | 12·5 | 4 | 4 | 3 | 0 | 1 | 1 |
| | 25 | 8 | 8 | 6 | 0 | 2 | 2 |
| | 50 | 8 | 8 | 6 | 0 | 2 | 2 |
| | 100 | 8 | 8 | 5 | 0 | 3 | 3 |
| | 200 | 4 | 4 | 4 | 0 | 0 | 0 |
| | All doses | 32 | 32 | 24[5] | 0 | 8[5] | 8[5] |

[1] Significantly different from syngeneic cell controls at same dose $P<0\cdot01$ (Fisher's exact test).
[2] Significantly different from syngeneic cell controls at same dose $P<0\cdot05$ (Fisher's exact test).
[3] Totals significantly different from syngeneic cell controls $P<0\cdot001$ ($\chi^2$ test).
[4] Totals significantly different from syngeneic cell controls $P<0\cdot005$ ($\chi^2$ test).
[5] Totals significantly different from syngeneic cell controls $P<0\cdot025$ ($\chi^2$ test).

TABLE II.—*Comparative Effects of Tumour Excision and Immunotherapy on the Incidence of Tumour Recurrence*

| Experimental group[1] | Average tumour size at resection (mm ± S.D.) | No. of recurrences | % "cured" | $P^3$ | Time of death after excision (days ± S.D.)[2] | $P^4$ |
|---|---|---|---|---|---|---|
| Untreated controls (Tu. D 0) | (8·9 ± 1·1) | (7/7) | 0 | | (55·6 ± 9·1) | |
| Tu. D 0—Surg. D 21 | 9·7 ± 1·5 | 7/10 | 30 | | 54·4 ± 5·7 | |
| Tu. D 0—GVHR D 14—Surg. D 21 | 8·5 ± 2·0 | 3/11 | 73 | NS | 76·7 ± 30·7 | NS |
| Tu. D 0—GVHR D 21—Surg. D 21 | 10·4 ± 1·5 | 4/8 | 50 | NS | 56·3 ± 7·3 | NS |

[1] Tu., Tumour; D, Day; Surg., Surgery; GVHR, graft-versus-host reaction.
[2] Data compiled on rats developing tumour recurrences.
[3] Probability based on Fisher's exact test. NS, Not significantly different from group with surgery alone.
[4] Probability based on Student's *t* test. NS, not significantly different from group with surgery alone.

same experimental group, then they were killed and their tissues examined histologically for tumour and signs of graft-versus-host disease. In Study 2 (Table II) tumours reaching a diameter of about 10 mm were resected on Day 21. In the case of graft-versus-host immunotherapy, $50 \times 10^6$ spleen cells from Wistar parental strain rats were injected intraperitoneally either on Day 14 or Day 21 respectively.

The effectiveness of the treatment was measured by the number of rats surviving more than 3 months. They are referred to as "cures" in the results.

In Study 1, the results of several experiments of the same kind were pooled and are summarized in Table I. In control rats, all 23 given tumour alone and 31 of 32 given tumour and syngeneic spleen cells died with widespread metastases; in the remaining one animal in the latter group the tumour grew to a diameter of 4 mm and then regressed completely. In those rats injected with spleen cells from the tumour susceptible Wistar parental strain there was a marked reduction of mortality from the tumour, particularly with doses of $25\times10^6$ and $50\times10^6$ cells. In some of these the tumour failed to take but in others it grew up to a diameter of 15 mm over a period of 4 weeks before regressing. Histological examination of the tumour inoculation site in these cases showed a small nodule of dense fibrous tissue containing necrotic or degenerate tumour cells. When a dose of $100\times10^6$ and $200\times10^6$ Wistar spleen cells was used, mortality was higher but this was mainly due to tumour growth; only one animal given $200\times10^6$ died from graft-versus-host disease. However, in animals in these groups dying with tumour the survival time was prolonged and, although massive tumours developed at the site of inoculation, there was no evidence of the usual secondary spread to other organs. Tumour regression was also associated with inoculation of spleen cells from the tumour resistant DA parental strain rats with all but the lowest cell dose but, in contrast with the Wistar cells, DA cells produced a high mortality from graft-versus-host disease so that there were few survivors in this group. In 2 rats given $25\times10^6$ DA cells tumours which grew up to a diameter of 25 mm over 4 weeks regressed, in one completely, while in the other rat the tumour regressed to a diameter of 7 mm, however, only to resume progressive growth, eventually killing the host. Regression of tumours also occurred on occasions with inoculation of allogeneic Lewis cells at most doses. These cells were less effective than parental Wistar strain cells when considering the number of survivors but they did not induce graft-versus-host disease so that there were more survivors than in the group given DA cells.

The preliminary results of the immunotherapeutic value of a graft-versus-host reaction in preventing the recurrence of tumours in surgically resected animals are summarized in Table II. The most effective regimen was obtained with application of a graft-versus-host reaction on Day 14, followed by surgery on Day 21. In the same regimen, when immunotherapy was delayed for 7 days the number of cures decreased. Immunotherapy alone on Day 14 was unable to cope with established tumours (unpublished observations).

The results show that injection of non-syngeneic spleen cells can in a limited range prevent the growth of a solid transplanted malignant tumour without causing death from graft-versus-host disease (Table I). Previous studies have shown that lymphoid cells of different inbred strains of rats vary considerably in the type of graft-versus-host reaction that they produce (Elkins, 1970) and in the present study lethal disease occurred consistently more often with parental DA cells than with equivalent doses of Wistar cells of the other parental strain, although this was not paralleled by any significantly greater effectiveness in inhibiting tumour growth. As has been shown previously with a methylcholanthrene induced sarcoma (Medzihradsky, 1969; Medzihradsky, Konikova and Novotna, 1973), increasing the dose of lymphoid cells beyond a certain level produces less inhibition of tumour growth as well as increased mortality from graft-versus-host disease.

Because the lymphoid cells that were most effective in producing tumour inhibition are syngeneic with the tumour, this effect cannot be explained as an allograft rejection reaction. We are probably witnessing an example of the allogeneic effect in which a variety of immunological and inflammatory responses are stimulated by foreign lymphoid cells (Elfenbein, Green and Paul, 1974; Osborne and Katz, 1973); this effect has previously been shown to inhibit progress of leukaemia (Katz *et al.*, 1972; Ellman *et al.*, 1972). The tumour used in the present study excites a weak host cellular immune response which does not obviously affect growth (Flannery *et al.*, 1973) but injection of BCG can induce host resistance to this tumour (Baldwin and Pimm, 1973) and perhaps the injection of parental or allogeneic lymphoid cells gives a similar augmentation of host anti-tumour immune response. In fact, preliminary results have shown under *in vitro* conditions that, shortly after the induction of a graft-versus-host reaction, spleen cells from tumour bearing F1 hybrids exert a significantly stronger cytotoxic effect upon tumour target cells of the tumour susceptible Wistar parental strain than spleen cells from animals given tumour alone (Rumma, in preparation). Furthermore, the possibility that the cancerous hosts were being stimulated to cope with metastases when a touch of graft-versus-host reaction was used in combination with surgical excision was indicated by the marked increase in cures obtained with this combination of therapies than when surgery alone was used. However, the fact that no significant prolongation of survival of the remaining rats was obtained in the combined therapy would indicate that survival might well have been dependent upon a critical ratio of metastases to immune response status. Nevertheless, the preliminary results strongly suggest that a controlled graft-versus-host reaction might become an effective anti-tumour adjunct in controlling residual tumour remaining after excision of the main mass.

We thank Professor R. C. Nairn for his advice and Professor R. W. Baldwin for the tumour and subline of Wistar rats used in this investigation. This work was supported by grants from the Anti-Cancer Council of Victoria and the Australian Research Grants Committee.

### REFERENCES

Baldwin, R. W. (1966) Tumour-specific Immunity against Spontaneous Rat Tumours. *Int. J. Cancer*, **1**, 257.

Baldwin, R. W. & Pimm, M. V. (1973) BCG Immunotherapy of Local Subcutaneous Growths and Post-surgical Pulmonary Metastases of a Transplanted Rat Epithelioma of Spontaneous Origin. *Int. J. Cancer*, **12**, 420.

Barnes, D. W. K., Loutit, J. F. & Neal, F. E. (1956) Treatment of Murine Leukaemia with X Rays and Homologous Bone Marrow. *Br. med. J.*, ii, 626.

Elfenbein, G. J., Green, I. & Paul, W. E. (1974) The Allogeneic Effect: Increased Cellular Immune and Inflammatory Responses. *J. Immun.*, **112**, 2166.

Elkins, W. L. (1970) Specific and Nonspecific Lymphoid Cell Proliferation in the Pathogenesis of GVHRs. *Transplantation*, **9**, 273.

Ellman, L., Katz, D. H., Green, I., Paul, W. E. & Benacerraf, B. (1972) Mechanisms Involved in the Antileukemic Effect of Immunocompetent Allogeneic Lymphoid Cell Transfer. *Cancer Res.* **32**, 141.

Fefer, A. (1973) Adoptive Tumor Immunotherapy in Mice as an Adjunct to Whole-body X-irradiation and Chemotherapy. A Review. *Israel J. med. Sci.*, **9**, 350.

Flannery, G. R., Chalmers, P. J., Rolland, J. M. & Nairn, R. C. (1973) Immune Response to a Syngeneic Rat Tumour: Development of Regional Node Lymphocyte Anergy. *Br. J. Cancer*, **28**, 118.

Katz, D. H., Ellman, Paul, W. E., Green, I. & Benacerraf, B. (1972) Resistance of Guinea Pigs to Leukemia Following Transfer of Immunocompetent Allogeneic Lymphoid Cells. *Cancer Res.*, **32**, 133.

Medzihradsky, J. (1969) On the Mechanisms of Tumour Facilitation and Tumour Inhibition Effect of the Local GVHR. *Neoplasma*, **16**, 3.

Medzihradsky, J., Konikova, E. & Novotna, L. (1973) On the Graft-versus-host Nature of the Allogeneic Effect in a Tumour Isograft System. *Neoplasma*, **20**, 607.

Osborne, D. P. Jr & Katz, D. H. (1973) The Allogeneic Effect in Inbred Mice. IV. Regulatory Influences of Graft-vs.-host Reactions on Host T Lymphocyte Function. *J. exp. Med.*, **138**, 825.

Rumma, J. & Davies, D. J. (1974) Accelerated Growth of a Strain Specific Rat Tumour Transplanted into F1 Hybrids. *Br. J. Cancer*, **30**, 582.

Santos, G. W. (1972) The Application of Marrow Grafts in Human Disease: Its Problems and Potential. *Contemp. Top. Immunobiol.*, **1**, 143.

Wigzell, K. (1961) Immunological Depression of Tumor Growth in F1 Hybrid/Parental Strain Systems. *Cancer Res.*, **21**, 365.

WOODRUFF, M. F. A. & SYMES, M. O. (1962) The Use of Immunologically Competent Cells in the Treatment of Cancer. Experiments with a Transplantable Mouse Tumour. *Br. J. Cancer*, **16**, 707.

WOODRUFF M. F. A., SYMES, M. O. & STUART, A. E. (1963) The Effect of Rat Spleen Cells on Two Transplanted Mouse Tumours. *Br. J. Cancer*, **17**, 320.